# Vertically infected *Aedes aegypti* excrete infectious arboviruses in saliva

**DOI:** 10.1186/s12915-026-02562-2

**Published:** 2026-02-25

**Authors:** Gladys Gutierrez-Bugallo, Elodie Calvez, Christelle Dollin, Yanet Martínez, Géraldine Piorkowski, Xavier de Lamballerie, Anubis Vega-Rúa

**Affiliations:** 1 Department of Vector Control, Center for Research, Diagnostic, and Reference, Institute of Tropical Medicine Pedro Kourí, PAHO-WHO Collaborating Center for Dengue and Its Control, Havana, 17100 Cuba; 2 Vector-Borne Diseases Laboratory, Environment and Health Research Department, Institute Pasteur of Guadeloupe, Les Abymes, Guadeloupe 97139 France; 3 Unité Des Virus Émergents (UVE: Aix-Marseille Univ, IRD 190, Inserm 1207, IRBA, Marseille, 13005 France; 4 Present Address: W. Harry Feinstone Department of Molecular Microbiology and Immunology, Malaria Research Institute, Bloomberg School of Public Health, Johns Hopkins University, Baltimore, MD USA

**Keywords:** Vertical transmission, Mosquito, Arboviruses, *Aedes aegypti*, Saliva, Dengue, Zika, Chikungunya

## Abstract

**Background:**

Epidemics of dengue (DENV), chikungunya (CHIKV), and Zika (ZIKV) viruses are primarily driven by transmission to humans via *Aedes aegypti* mosquitoes. In addition to this horizontal route, *Ae. aegypti* can also transmit these viruses to their offspring through vertical transmission. In this study, using a field-derived *Ae. aegypti* population, we assessed vertical transmission of the three viruses, and we assessed whether female mosquitoes maternally infected can excrete infectious virus in their saliva—a key requirement for subsequent transmission to humans.

**Results:**

We detected infectious CHIKV and ZIKV in the saliva of 15% and 11% of daughters obtained from infected mothers, respectively. In the case of DENV-1, 14% of female offspring had infectious virus in their heads, although saliva samples were negative, meriting further investigation. Moreover, pooled male progeny was found positive for CHIKV and ZIKV.

**Conclusions:**

These findings suggest that vertical transmission may be an underestimated mechanism which provides a potential route by which *Ae. aegypti* can become infective independently of biting a viremic host, thereby contributing to arbovirus persistence and spread. The presence of infected male progeny also demonstrates that vertically transmitted viruses can persist in mosquito populations even in individuals that do not blood-feed.

**Supplementary Information:**

The online version contains supplementary material available at 10.1186/s12915-026-02562-2.

## Background

*Aedes*-borne viruses infect humans through the bite of an infective female mosquito following a mosquito-vertebrate-mosquito transmission pattern, also called horizontal transmission (HT) [1]. In the case of dengue (DENV), chikungunya (CHIKV), and Zika (ZIKV) viruses, this HT is mainly ensured in tropical settings by the anthropophilic *Aedes aegypti* mosquito [2]. However, an “alternative” route is known to occur inside the vector populations: the vertical transmission (VT), in which the virus is transmitted from infected parents to their offspring [1]. Although arbovirus HT is the most recognized source of mosquito infections, VT is considered an evolutionary mechanism for viral persistence in nature during adverse periods for HT, and one of the likely explanations for virus re-emergence [1].

Parent-to-offspring transmission has been documented in several mosquito–virus systems [3]. This process is presumed to occur when the virus, following infection of the female mosquito, invades the ovarian and other reproductive tissues establishing the mechanistic basis for VT [4–6]. While some authors consider VT a relatively infrequent and meaningless phenomenon [7, 8], numerous reports from arbovirus endemic regions such as India [9, 10], the Philippines [11], Mexico [12], Cuba [13], and Brazil [14, 15] suggest that VT may favor both the sustenance and co-circulation of arboviruses in those settings [9, 16]. In *Aedes* mosquitoes, desiccation-resistant eggs enable survival during unfavorable conditions, allowing vertically transmitted viruses to persist even when adult females cannot sustain horizontal transmission [1]. When favorable conditions return, the hatching of infected larvae may promote the re-establishment of both mosquito and viral populations, emphasizing the potential role of VT in arbovirus maintenance [17]. However, VT alone does not guarantee epidemiological impact [18]: to contribute to arbovirus circulation, VT must produce vertically infected females that, as soon as they emerge, are able to excrete infectious virus in their saliva and infect a new human.

To achieve a human infection through HT, the first transmission of a virus by *Ae. aegypti* can only occur after completion of the extrinsic incubation period (EIP), which corresponds to the time required for a mosquito to become infective after the ingestion of the virus. Thus, the virus ingested must infect and replicate in mosquito midgut epithelial cells, disseminate to hemocoel, colonize the insect salivary glands and finally, be expelled in the saliva during the next blood meal [19]. In *Ae. aegypti*, the EIP ranges from 7 to 10 days for DENV [20] or ZIKV [21] and from 2 to 3 days for CHIKV [22]. Infective *Ae. aegypti* females resulting from vertical infections could arise as a novel paradigm of horizontal transmission, since no previous infected blood meal nor EIP would be needed for that newly emerged female to transmit the virus.

Although few studies have investigated the potential of VT in *Ae. aegypti* to initiate HT [18, 23–25], these have primarily used long-term laboratory colonies, which do not accurately represent field conditions, or have focused solely on the detection of viral RNA. To date, no published study has demonstrated that non-colonized, vertically infected *Ae. aegypti* females can excrete infectious viral particles in their saliva immediately after emergence. Given the significant global public health burden posed by DENV, CHIKV, and ZIKV, as well as the limited understanding of how VT contributes to viral maintenance and outbreak dynamics, this study aimed to assess the transmission potential of *Ae. aegypti* females derived from field-collected eggs (F₁ generation) obtained in areas with previous arbovirus circulation. We experimentally evaluated vertically infected females for the presence of infectious DENV-1, CHIKV, and ZIKV particles in their saliva to determine whether VT can produce infective mosquitoes capable of initiating HT.

## Results

**Fig. 1:**
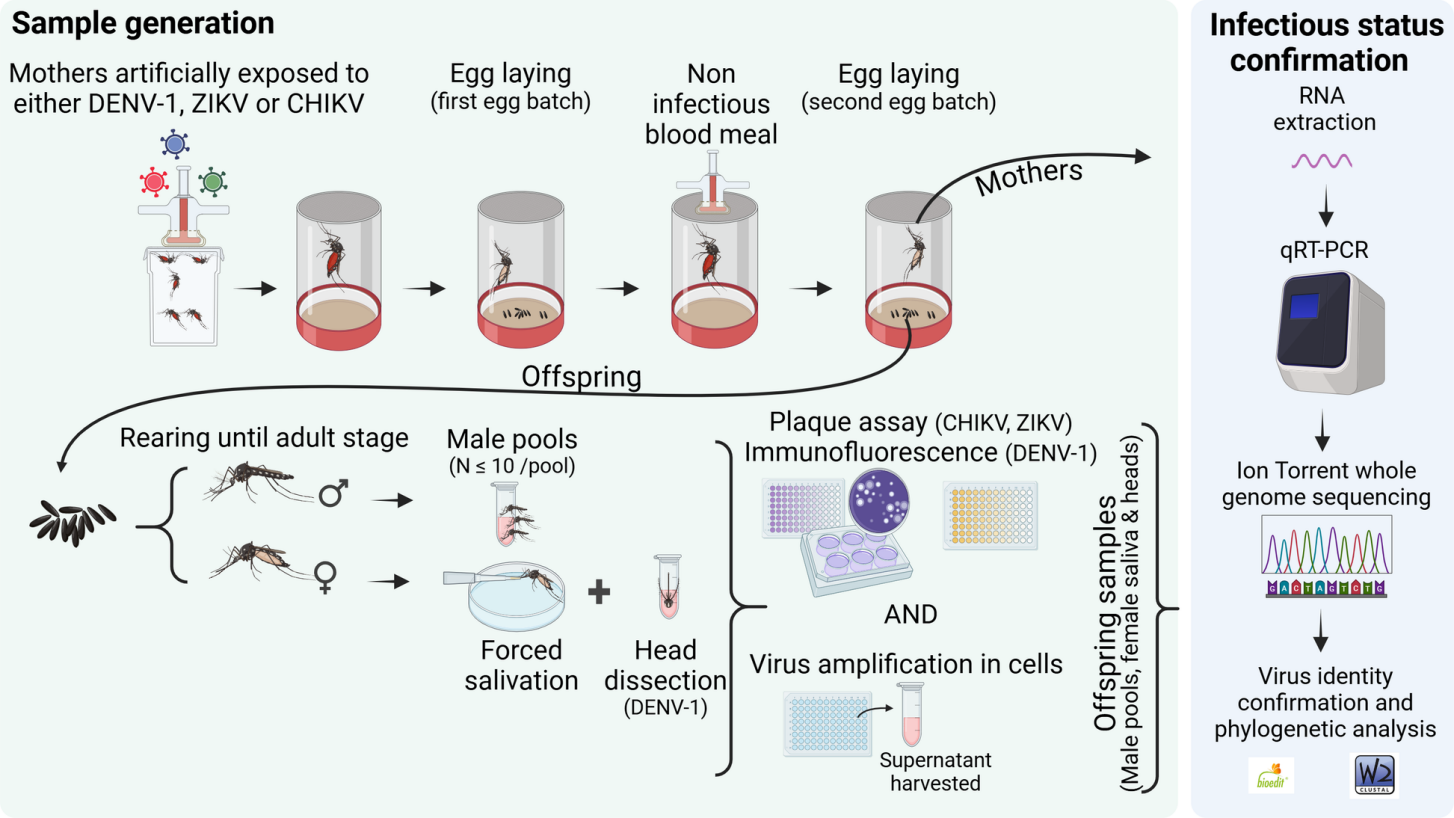
Workflow of vertical transmission assays conducted with *Aedes aegypti* from Havana, Cuba (F1 generation) infected with dengue virus type 1, chikungunya, or Zika viruses. A general overview of each vertical transmission assay is illustrated. Daughter’s head examination was conducted exclusively for dengue-1 virus vertical transmission assay. Created in BioRender. VEGA RUA, A. (2026) https://BioRender.com/v82a781

VT assays allowed the obtention of offspring from *Ae. aegypti* females from Havana, Cuba, that had been individually exposed to DENV-1, CHIKV, or ZIKV (Fig. 1) (Additional file 1: Table 1). The following VT parameters were estimated for each assayed mosquito population/virus pair: (i) vertical transmission rate to saliva (VTR-S), calculated as the proportion of infected mothers from which at least one infective daughter (with infected saliva) was obtained and (ii) filial infective rate (FIR-S), representing the proportion of infective progeny (i.e. daughters with positive saliva) from infected mothers.

### Vertically infected *Ae. aegypti* females can excrete infectious CHIKV and ZIKV in their saliva since they emerge

**Fig. 2:**
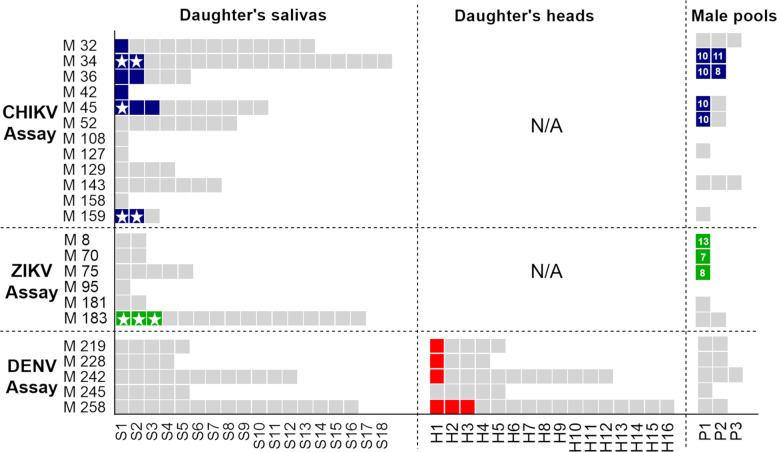
Heat map displaying vertically infected and uninfected progeny obtained from each infected mother in vertical transmission assays conducted with non-colonized *Aedes aegypti* from Cuba and dengue virus type 1 (DENV-1), chikungunya (CHIKV), and Zika (ZIKV) viruses. Each gray box corresponds to a different mosquito progeny. Samples infected with CHIKV, ZIKV, or DENV-1 viruses are colored in blue, green, or red, respectively. M: mother, S: daughter’s saliva, H: daughter’s head, P: male pools, N/A: not assayed. Stars within the squares indicate saliva samples that tested positive by direct titration or exhibited cytopathic effect (CPE) following virus amplification in cell culture. Numbers above the colored squares representing male pools indicate the total number of males tested in each pool

Our data demonstrated the presence of infectious CHIKV and ZIKV particles in saliva from vertically infected daughters (Table 1, Fig. 2). Regarding CHIKV, out of 12 CHIKV-infected mothers, seven transmitted the virus vertically. A total of 72 newly emerged females were recovered, and 11 saliva samples collected from them were found positive for CHIKV. Of these, five were detected by direct saliva titration and seven by real-time RT-PCR (RT-qPCR) after virus multiplication in cells (Table 1, Fig. 2). The viral load in daughters’ saliva, estimated by direct titration, ranged from 1.2 to 3.3 log_10_ plaque-forming units (pfu)/saliva. The CHIKV filial infective rate (daughters with positive saliva) represented 15% of the newly emerged females recovered from infected mothers (FIR-S = 15%). They came from six infected mothers, thus representing a VTR-S of 50% (Table 1). The CHIKV full genome sequencing (11,226 bp) demonstrated that the sequence obtained from two mothers (GenBank accession numbers: OR488123 [26], OR488124 [27]) and saliva from daughters (GenBank accession number: OR488125 [28]) shared more than 99% of identity with the sequence from the viral supernatant used for the oral infection (GenBank accession number: OR488122 [29]).

**Table 1:** Vertical transmission data and parameters estimated after oral exposure of *Aedes aegypti* from Cuba (F1 generation) to chikungunya, Zika, or dengue-1 viruses

	**Description**	**CHIKV**	**ZIKV**	**DENV-1**
Samples obtained	Number of mothers exposed to viruses (1st GC)	34	23	43
	Number of mothers at 2nd GC	12	6	12
	Number of infected mothers *	12	6	5
	Mothers that vertically transmitted the virus at 2nd GC	7	4	4
	Saliva samples of daughters from infected mothers	72	28	42
	Daughters w/ infected saliva	11	3	0
	Daughters w/ infected head**	NA	NA	6
	Infected male pools	6	3	0
	Families w/ both infected saliva and male pools	3	0	0
Vertical transmission parameters % (95% CI)	VTR-S	50 (25–75)	17 (3–56)	0
	VTR-H	NA	NA	80 (38–96)
	FIR-S	15 (9–25)	11 (4–27)	0
	FIR-H	NA	NA	14 (7–28)

*GC* gonotrophic cycle. *VTR-S* vertical transmission rate to saliva: proportion of infected mothers from which at least one infective daughter (with infected saliva) was obtained. *VTR-H* vertical transmission rate to head: proportion of infected mothers from which at least one daughter that disseminated the virus (virus in the head) was obtained. *FIR-S* filial infective rate: proportion of infective offspring (daughters with positive saliva) from infected mothers. *FIR-H* filial infection rate to head: proportion of infected offspring (daughters with positive heads) from infected mothers. *Infection in mothers was detected by RT-qPCR. **Infection in daughter heads was detected by RT-qPCR after amplification in C6/36 cells. *NA* not assayed

Regarding ZIKV, VT assays yielded six infected mothers that laid eggs at 2nd GC, of which four vertically transmitted the virus (Table 1). Among the 28 salivas collected from newly emerged daughters, only three (belonging to the same mother) were found positive for ZIKV after inoculation onto Vero cells by both cytopathic effect (CPE) and RT-qPCR on the supernatant (Fig. 2). The pair *Ae. aegypti*-ZIKV displayed a VTR-S of 17% and a FIR-S of 11%. No ZIKV nucleotide sequence was obtained from the positive salivas.

### Vertically infected *Ae. aegypti* females harbored DENV-1 infectious particles in their heads

VT assays conducted with DENV-1 and the same F1 field *Ae. aegypti* population were more similar than for CHIKV and ZIKV (Fig. 1). The infectious status of heads and saliva from daughters obtained at the second gonotrophic cycle (2nd GC) from DENV-exposed mothers was determined by viral titration, RT-qPCR, and sequencing (Fig. 1). Saliva analysis conducted on the 42 daughters obtained from infected mothers did not reveal any positive sample for DENV-1, neither by direct saliva titration nor real-time RT-PCR after virus amplification on C6/36 cells. However, the heads of six female offspring were positive by RT-qPCR after virus amplification on C6/36 cells. Consequently, we determined the vertical transmission rate to head (VTR-H) as the proportion of infected mothers from which at least one daughter had a positive head. Similarly, the filial infection rate to head (FIR-H) represented the proportion of daughters with positive heads.

For DENV-1, 12 mothers laid eggs at 2nd GC of which five were found infected. Of these, four transmitted vertically the virus to their progeny (Table 1). We did not detect DENV-1 in daughter heads by direct titration. However, RT-qPCR analysis on daughter heads (with uninfected saliva) demonstrated the presence of DENV-1 in six samples among the 42 tested after inoculation onto C6/36 cells, representing a VTR-H of 80% and a FIR-H of 14% (Table 1, Fig. 2). Full genome sequencing could not be performed from those daughter heads, but short nucleotide DENV-1 fragments were obtained (248 bp, 187 bp, 205 bp, 244 bp, and 233 bp; Additional file 2: Table 2) and demonstrated > 98.8% identity with viral supernatant used for the oral infection (GenBank accession number: OR486055).

Finally, no statistically significant differences in VTR-S were observed among the viruses tested (*P* = 0.1369). However, a significant difference in FIR-S was found between CHIKV- and DENV-1-infected progeny (*P* = 0.0066).

#### Male progenies exhibit vertical infection

Pools up to 10 male offspring from the 2nd GC were also examined by RT-qPCR and sequencing after exposing the F1 field population *Ae. aegypti* females to DENV-1, CHIKV, or ZIKV (Fig. 1).

After examining 16 male offspring pools (*N* = 159), obtained from CHIKV-exposed females, we found that 33% of the infected mothers transmitted vertically the virus to their male offspring, while at least 5% of that male progeny was positive for CHIKV, considering that at least one male mosquito was infected in the pool (Fig. 2). We found coincidence among CHIKV positive male pools and daughters (saliva) in 43% of mothers (Table 1).

Regarding ZIKV VT assay, six male pools (*N* = 57) were tested (Fig. 2). The mothers who transmitted the virus to males (*N* = 3 pools) did not lead to any infective daughter (no positive saliva detected) (Table 1, Fig. 2). On the other hand, we did not detect any DENV-1 positive male pool among the 10 gathered (*N* = 90) from infected mothers (Fig. 2). Differences in infection levels between male and female progeny may be attributed to the limited sample size and the use of pooled samples for males rather than individual testing. Given that egg infection likely occurs prior to fertilization, there is no biological basis to expect a difference in infection rates between male and female offspring.

## Discussion

VT of DENV-1, CHIKV, and ZIKV in *Ae. aegypti* has been documented naturally and experimentally in many places around the world [4, 9, 10, 12–15, 30]. Nevertheless, the epidemiological significance of VT remains controversial for most arboviruses, since maintenance in the mosquito population through VT alone is not sufficient to start another outbreak. To horizontally infect a human host, the virus must be present in the saliva of female mosquitoes [18, 31, 32]. Detection in other tissues (e.g., head, legs, or excreta), therefore, does not necessarily indicate transmission potential because salivary-gland infection and escape barriers can prevent viral release into the saliva [33, 34].

Few studies experimentally demonstrated that vertically infected females could transmit viruses horizontally involving diverse mosquito—virus combinations: La Crosse (LACV)-*Aedes* (*Protomacleaya*) *triseriatus* [35–38], California encephalitis-*Aedes (Ochlerotatus) dorsalis* [39], and ZIKV-*Aedes (Stegomyia) albopictus* [40]. Nevertheless, evidence of infectious DENV-1, CHIKV, nor ZIKV in saliva of vertically infected *Ae. aegypti* females had not been reported so far. This study demonstrated, for the first time, the infective ability of newly emerged *Ae. aegypti* females from a field population (F1 generation) to transmit CHIKV and ZIKV through the detection of infectious viral particles in their saliva. We also found DENV-1 in offspring heads. Viral nucleotide sequences obtained from saliva and head samples of female progeny confirmed viral identity and our diagnosis.

CHIKV VT has often been debated as inconsistent findings have been observed under laboratory conditions [41–44]. These discrepancies are likely attributable to differences in experimental methodologies such as the maternal infection route, viral detection assays, gonotrophic cycle analyzed, or developmental stage tested, as well as to the mosquito and viral strains used [1]. To the best of our knowledge, we demonstrated for the first time that female offspring vertically infected with CHIKV can expel infectious particles via their saliva. A tangible contribution of VT to HT would be the frequencies and proportions in which infective daughters are produced by the infected mothers. In the present study (i) half of CHIKV-infected mothers gave rise to at least one female offspring with positive saliva (VTR-S = 50%) and (ii) 15% of the daughters from infected mothers were infective (FIR-S = 15%, Table 1). Even if larger sample sizes or different mosquito–viral strain combinations could yield different estimations, our results highlight the potential role of CHIKV VT in generating daughters capable of transmission and suggest a mechanism that may enhance viral circulation within mosquito populations. In this scenario, infection could originate not only from biting a viremic host but also from an infected mother, potentially facilitating the virus’s episodic reintroduction into a HT cycle. Vertically infected females may further sustain virus persistence during inter-epidemic periods through desiccation-resistant eggs [1, 17]. The magnitude of these contributions is likely modulated by strain- and environment-specific factors [1, 3, 45] that deserve further investigation.

Regarding ZIKV, VT by *Ae. aegypti* mosquitoes in the lab has been already demonstrated [4, 18, 24, 25, 46], and ZIKV genomes have been previously found by RT-qPCR in salivary glands [25] and saliva [18, 24] of females vertically infected in the laboratory. However, the mere detection of ZIKV genomes does not prove the presence of infectious viral particles in saliva [18]. In our study, we confirmed the presence of infectious ZIKV in the saliva of vertically infected, uncolonized *Ae. aegypti* females. We also estimated a FIR-S of 11%, that agrees with previous findings that report ranges from 2 to 17% in salivary glands [25] or saliva [18, 24] of vertically infected *Ae. aegypti* females. We acknowledge that only a small number of ZIKV-infected mothers were obtained (N = 6), and that infectious virus was detected in the offspring of just one mother, which compromises the reliability of the VTR-S estimated and prevents us from accurately assessing the extent of this phenomenon. Nevertheless, when considered together with earlier studies, these results clearly indicate that a subset of daughters from ZIKV-infected mosquitoes harbor infectious virus in their saliva, suggesting that VT can produce females potentially capable of initiating HT.

The EIP of ZIKV in *Ae. aegypti* has been estimated around 7 to 10 days [47–49] and may exceed 14 days for certain ZIKV genotypes [50]. Since newly emerged females were able to expel infectious virus immediately after emergence, this represents a significant reduction in EIP for 11% of daughters (FIR-S) according to our observations. Vector competence studies from diverse *Ae. aegypti* populations have reported relatively low ZIKV transmission rates (often below 25% at 14 days post-exposure) [47–50], which contrasts with the virus’s rapid global spread [48]. While large naïve human populations and high mosquito densities have been recognized as major drivers of this expansion [49], our findings and previous evidence [4, 18, 24, 25, 46], suggest that VT could play a role in contributing to ZIKV dissemination. Vertically transmitted ZIKV reaching the saliva of newly emerged females may accelerate infectiousness by effectively shortening the EIP, thereby offering an additional route that could facilitate the virus’s rapid spread. Future modeling studies could help quantify the extent to which VT could contribute to these transmission dynamics.

Regarding DENV, Mourya and colleagues [51] previously showed that an isofemale *Ae. aegypti* line highly susceptible to DENV-2 produced infected progeny capable of transmitting the virus when allowed to probe on bovine albumin phosphate [51]. However, colonized or highly susceptible laboratory-selected mosquito lines are generally more permissive to infection and dissemination than field-derived mosquitoes [52]. In our study, DENV-1 was not detected in the saliva of vertically infected daughters, yet this does not necessarily preclude the occurrence of VT leading to potential infectivity. The same saliva collection procedure successfully detected infectious CHIKV and ZIKV, but methodological and biological factors could still have contributed to DENV-1 non-detection. For instance, (i) in vitro saliva collection methods are known to underestimate both the number of positive samples and viral quantities [53], and (ii) low viral loads may have limited detection sensitivity [18] (i.e., 15 CHIKV saliva samples with Ct > 34). Moreover, the limited number of DENV-1–infected mothers obtained in our assay may have constrained the probability of detecting infected offspring. Importantly, detection of replicative DENV-1 in the heads of 14% of female offspring after multiplication in C6/36 cells confirms the presence of infectious virus. Although DENV detection in the heads of horizontally infected mosquitoes is frequently used as a proxy for potential transmission capability in vector competence studies [54–57], this remains to be demonstrated for vertically acquired infections, as viral tropism following vertical infection remains largely uncharacterized.

Limited knowledge currently exists regarding potential vertical infection barriers. Previous works using the pair *Aedes triseriatus*—LACV revealed multiple organs or germ layers as sources of infection in developing mosquito larvae and pupae [32]. In both immature stages, LACV was found in the alimentary tract (including salivary glands) and other tissues. It is expected that through transovarial transmission (TOT) (one of the two mechanisms proposed for VT), the virus infects germinal tissues in female mosquitoes, therefore achieving higher VT rates and extensive dissemination [58]. Flaviviruses VT is thought to occur by trans-egg mechanism, considered less efficient than TOT [59]. Nevertheless, patterns consistent with stabilized infections that reach high VT rates were reported for DENV-1 in *Ae. albopictus* [60] as well as for DENV-3 [61] and ZIKV [46] in *Ae. aegypti,* suggesting that an efficient dissemination in mosquito offspring could be expected as well [1]. Although some publications question the occurrence and epidemiological significance of VT of DENVs [7, 8, 62], the growing number of natural VT reports for these viruses [11, 63–65], as well as experimental demonstration for occurrence [30, 51, 61, 66] are in line with our findings and hypothesis.

Taken together, previous evidence and our results indicate that VT in *Ae. aegypti* may play a complementary role in arbovirus spread, linking vertical and HT routes with potential epidemiological consequences. Vertically infected females may be capable of transmitting virus immediately upon emergence, bypassing the need for an initial infectious blood meal and the EIP. This could effectively extend the “infectious period” at the population-level and potentially accelerate virus transmission dynamics [18]. VT may also support inter-epidemic maintenance, allowing viruses to persist in mosquito populations when human transmission chains are interrupted [1, 17]. A modeling study indicates that higher VT rates can increase the basic reproductive number (${R}_{0}$), particularly when ${R}_{0}$ approaches 1, suggesting VT could help trigger outbreaks that would otherwise be marginal [67]. Further work is needed to determine whether vertically infected progeny has a shorter functional EIP or altered vector competence compared with horizontally infected mosquitoes, to better quantify the epidemiological impact of VT.

Six CHIKV-infected and three ZIKV-infected male pools were detected (Table 1; Fig. 2), highlighting a potential contribution to venereal transmission, as previously demonstrated for several arboviruses [30, 68, 69]. Though Sanchez-Vargas et al. [30] demonstrated that vertically infected males can transmit the virus venereally, further studies should address the actual contribution of venereal transmission in arbovirus transmission dynamics. Moreover, the detection of infected male pools underscores that viruses can persist independently of blood meals, supporting the notion that vertically infected males may contribute to arbovirus maintenance in mosquito populations. VT remains relatively underrecognized in mosquito-borne disease epidemiology [3], in part due to technical constraints related to assay sensitivity in both field and laboratory contexts [31], although its occurrence has been widely documented [3, 70]. Furthermore, VT assays are extremely time consuming and the resulting sample sizes are often impacted by the small number of mosquito mothers that survived through their second gonotrophic cycle and laid eggs [8, 71]. Depending on the study goals of VT research, these limitations could be partially mitigated by alternative approaches, such as intrathoracic virus injection to bypass midgut barriers and accelerate viral dissemination, which can reduce the time and effort required for VT assays. More sensitive viral detection capacities would allow more accurate VT estimations. As sufficient viral load is crucial to guarantee host infection, future studies should address the infectivity of vertically infected saliva using appropriate animal models [72].

## Conclusions

Our findings highlight that VT may be an underestimated mechanism which provides a potential route by which *Ae. aegypti* can become infective independently of biting a viremic host, thereby contributing to arbovirus persistence and spread. Hence, VT should be considered in vector control programs by sustaining interventions during both epidemic and inter-epidemic periods, with particular attention to breeding sites and eggs that may harbor infection. This approach can better account for the role of VT in the long-term persistence and management of *Aedes*-borne viruses [3]. Given the anthropophilic and domestic behavior of *Ae. aegypti* and the significant public health impact of arboviruses, social communication campaigns should emphasize that *Ae. aegypti* eggs may serve as potential reservoirs for virus persistence, encouraging continuous community engagement in eliminating breeding sites even in inter-epidemic seasons.

## Methods

### Mosquito population

F0 *Ae. aegypti* eggs were collected using ovitraps in two localities of Havana, Cuba (23.094494, 82.365780 and 23.06199, 82.353201) and hatched in dechlorinated tap water. Species identification was performed at the adult stage using morphological keys [73]. Larvae were reared at a density of 150–200 larvae/liter, under laboratory conditions (28° ± 1 °C, 80% relative humidity, and 12 h:12 h light:dark cycle) and fed with rabbit food pellets (GMA, Guadeloupe). Water and diet were renewed every 3–4 days. Adults were kept in cages under the same laboratory conditions described above and supplied ad libitum with a 10% sucrose solution. To assess the infectious status of the F0 adults derived from field-collected eggs (*N* = 1000) and rule out the presence of DENV in these samples, an RT-qPCR using Lightmix Modular Zika (Roche, Panama) and Lightmix Modular Dengue (Roche, Panama) kits was performed according to the manufacturer’s instructions. F0 adults found negative were fed twice per week using artificial blood meal and a Hemotek system (Hemotek Ltd., Blackburn, UK) to obtain F1 eggs that were used for vertical transmission assays.

### Viral strains and cell cultures

Infections were conducted with two *Flavivirus* (DENV and ZIKV) and one *Alphavirus* (CHIKV) (Additional file 1: Table 1). DENV-1 and CHIKV strains were isolated from patients in Guadeloupe. ZIKV strain was provided by the Emergence Virus Unit (Marseille) via the initiative “European Virus Archive goes global” (EVAg). Virus stocks were generated using a multiplicity of infection of 0.1, after passages on Vero cells (ATCC, ref. CCL-81) for CHIKV and ZIKV, and C6/36 cells (ATCC, ref. CRL-1660) for DENV-1. Supernatants were collected and the viral titer was estimated using serial tenfold dilutions on Vero cells expressed as median tissue culture infectious dose (TCID_50_/mL) for CHIKV and ZIKV. Because DENV-1 does not always produce CPE in mammal cells, we estimated the viral titers by focus fluorescent assays on C6/36 cells and expressed in focus-forming units (FFU/mL). The viral stocks obtained were kept at − 80 °C until use.

### Vertical transmission assays

Seven- to 10-day-old female mosquitoes were fed with an infectious blood-meal at a viral concentration of 10^7^ Tissue Culture Infectious Dose _50_ (TCID_50_)/mL for CHIKV and ZIKV, and 10^7^ FFU/mL for DENV-1 using an Hemotek system as described in [35]. Fully engorged females were individually maintained in 50 mL Falcon tubes containing moistened filter paper as an oviposition substrate and covered with a net. Females were kept under the same conditions used for mosquito rearing in a climatic chamber (Memmert, Schwabach, Germany). After recovery of the first egg batch (between days 6 and 10), a second noninfectious blood meal was offered to each female. Eggs from the 2nd GC were collected and mothers were stored at − 80 °C (Fig. S1).

Eggs from 2nd GC collected from each mother were stored for 72 h to guarantee proper embryogenesis and then they were separately hatched and reared under the controlled conditions described above. Pupae were individually maintained in 5 mL tubes containing a solid substrate to provide a resting surface for adults after emergence. Tubes were monitored daily, and newly emerged females were cold-anesthetized. Saliva from individual daughters was subsequently collected as described by Gutiérrez-Bugallo and colleagues [43]. Saliva samples were then stored at − 80 °C in Leibovitz L15 medium for DENV-1 and in Dulbecco’s modification of Eagle’s medium (DMEM) for ZIKV and CHIKV to a final volume of 50 μL. In addition, pools containing up to ten male offspring from the same mother, the entire mothers, and heads of daughters from DENV-1-exposed mothers were homogenized in 300 μL of culture media supplemented with 2% FBS (Leibovitz L15 for DENV-1 and DMEM for ZIKV and CHIKV). All the homogenates were centrifuged at 10,000 × *g* for 5 min and the supernatants were frozen at − 80 °C until use.

### Viral detection

Two complementary approaches were employed to assess viral infection in mosquito samples. Virus titration was conducted using plaque assays to detect viable viral particles, providing evidence of active viral replication. Viral genome detection was performed by RT-qPCR to rapidly confirm viral identity and detect the presence of viral RNA, including cases where infectious particles were below the detection threshold of plaque assays.

#### Virus titration

For ZIKV and CHIKV, 30 μL of saliva collected from 2nd GC’s daughters was added to 270 μL of DMEM to obtain a final volume of 300 μL. The mixture was then inoculated onto monolayers of Vero cells in 6-well plates, overlaid with 2% agarose, and incubated at 37 °C, 5% CO_2_ for 7 days (ZIKV) and 3 days (CHIKV). Monolayers were then stained with a solution of crystal violet (0.2% in 10% formaldehyde and 20% ethanol). Evidence of viral particles was assessed by detection of CPE and titers were expressed as log pfu/saliva. For DENV-1, 40 μL of saliva collected from daughters was diluted in L15 medium to a final volume of 50 μL and then inoculated onto C6/36 cells in 96-well plates as well as 50 μL of head homogenates. After incubation at 28 °C for 5 days, the plates were fixed with 10% formaldehyde, then stained using mouse anti-dengue complex monoclonal, clone D3-2H2-9–21 (Merck KGaA, Germany) as primary antibody and Alexa Fluor 488 goat anti-mouse IgG as the second antibody (Life Technologies, Carlsbad, USA).

Because CPE alone does not confirm viral identity, we also tested progeny samples using RT-qPCR. Given the limited sample volume remaining after direct titration, prior viral amplification was performed by inoculating 96-well plates containing monolayers of Vero cells (for ZIKV and CHIKV) or C6/36 cells (for DENV-1) with saliva and head samples from female progeny, as well as homogenized male pools. Cells were maintained under the same conditions as previously described. Following incubation, supernatants were collected and stored at –80 °C for subsequent RNA extraction and real-time RT-qPCR analysis. Crystal violet staining was applied to Vero cell monolayers to assess CPE.

#### Viral genome detection

RNA was extracted from mothers, amplified saliva and head samples from daughters, and male pools using the Nucleospin RNA kit (Macherey–Nagel GmbH, Düren, Germany) according to the manufacturer’s instructions. RT-qPCR was performed in samples previously amplified in cell cultures to detect viral genomes of ZIKV, CHIKV, and DENV-1 using the primers/probes published by [74–76], respectively. RT-qPCR was conducted using the Superscript® III Platinum® One-Step RT-qPCR kit instructions (Invitrogen, Carlsbad CA, USA) and the Applied Biosystems 7500 real-time PCR system instrument (Applied Biosystems, Foster City, CA, USA). The thermal profile used was the following: 30 min at 50 °C for reverse transcription, 10 min at 95 °C for reverse transcriptase inactivation and DNA polymerase activation, 45 cycles at 95 °C for 15 s, and 31 s at 58 °C. In each case, samples were considered positive if amplification was detected at or below a cycle threshold (Ct) of 35 for ZIKV, 34 for CHIKV, and 39 for DENV-1. These Ct thresholds were selected based on standard curves of infectious particles-genomes performed for each virus (Additional file 3: Fig. S1). Samples with Ct lower than 32, 33, and 35 were selected for CHIKV, ZIKV, and DENV-1 sequencing, respectively.

### Viral sequencing

#### Viral amplification

For DENV, nine overlapping amplicons were produced using primers described in [77]. For CHIKV [78] and ZIKV, eight and twelve overlapping fragments were produced, respectively, as described in Additional file 4: Table 3 and Additional file 5: Table 4. Amplification was performed using 3 µL of RNA, 500 nM of each primer (Additional file 4: Table 3 and Additional file 5: Table 4) and the SuperScript™ IV One-Step RT-PCR System kit (Thermo Fisher) (12.5 µL of 2X Platinum™ SuperFi™ RT-PCR Master Mix and 0.5 µL of SuperScript™ IV RT Mix) in a 25 µL final volume. The thermal profile used was 2 min at 50 °C for reverse transcription, 2 min at 98 °C, 40 cycles consisting of denaturation at 98 °C, 10 s; hybridisation at 56 °C, 10 s; and elongation at 68 °C, 2 min 30 s ending with final elongation at 68 °C, 5 min. PCR products were pooled in equimolar proportions after purification using Nucleofast PCR Plate kit (Macherey Nagel).

#### Viral sequencing

After quantification (Qubit® dsDNA HS Assay Kit and Qubit 4.0 fluorometer (ThermoFisher)), amplicons were sonicated (Bioruptor, Diagenaode) into 250 bp long fragments. Libraries were built adding to fragmented DNA barcode for sample identification and primers with Ion Plus Fragment Library Kit using AB Library Builder System (ThermoFisher). To pool equimolarly the barcoded samples, a real-time PCR quantification step was performed using Ion Library TaqMan™ Quantitation Kit (Thermo Fisher). Next steps included an emulsion PCR of the pools and loading on 530 chip performed using the automated Ion Chef instrument (ThermoFisher) followed by sequencing using the S5 Ion torrent technology (Thermo Fisher) according to manufacturer’s instructions. Consensus sequence was obtained after trimming of reads (reads with quality score < 0.99, and length < 100 bp were removed and the 30 first and 30 last nucleotides were removed from the reads) and mapping of the reads on a reference (inoculum strain) using CLC genomics workbench software v.22.0.1 (Qiagen). Parameters for reference-based assembly consisted of match score = 1, mismatch cost = 2, length fraction = 0.5, similarity fraction = 0.8, insertion cost = 3, and deletion cost = 3. A de novo contig was also produced to ensure that the consensus sequence was not affected by the reference sequence. Full genome sequences obtained from offspring were submitted to GenBank and the partial sequences are displayed in Table S4. Their homology with parental strains used for the artificial infections was determined using identity matrix generated by BioEdit 7.2.5 [79] to determine the proportion of identical residues between the different sequences.

### Data analysis

Vertical transmission parameters for each assayed pair mosquito population/virus were estimated. Fisher’s exact test was performed using GraphPad Prism version 10.5.0 to compare VTR-S and FIR-S across viruses.

Regarding female-to-offspring transmission:

- Vertical transmission rate to saliva (VTR-S): proportion of infected mothers from which at least one infective daughter (with infected saliva) was obtained.
- Vertical transmission rate to head (VTR-H): proportion of infected mothers from which at least one daughter that disseminated the virus (virus in the head) was obtained; only estimated for DENV virus.

In terms of infected offspring proportions:

- Filial infective rate (FIR-S): Proportion of infective progeny (i.e., daughters with positive saliva) from infected mothers.
- Filial infection rate to head (FIR-H) was calculated using daughters with positive heads, only for DENV virus.

## Supplementary Information

Additional file 1: Table 1. Features of Zika, chikungunya, and dengue virus type 1 viral strains.

Additional file 2: Table 2. Nucleotide DENV-1 fragments obtained from vertically infected *Aedes aegypti.*

Additional file 3: Fig. S1. Standard curves for dengue virus type 1, chikungunya, and Zika viruses.

Additional file 4: Table 3. Sequences of primers used for specific amplification of CHIKV.

Additional file 5: Table 4. Sequences of primers used for specific amplification of ZIKV.

## Data Availability

All data generated or analyzed during this study are included in this published article, its supplementary information files and publicly available repositories. The nucleotide sequences generated in this study have been deposited in GenBank under accessions OR48812229 [29], OR48812326 [26], OR48812427 [27], and OR48812528 [28]. Any further information required to reanalyze the data/conclusions reported in this study is available from the lead contact upon request.

## Abbreviations

DENV: Dengue virus
CHIKV: Chikungunya virus
ZIKV: Zika virus
HT: Horizontal transmission
VT: Vertical transmission
EIP: Extrinsic incubation period
VTR-S: Vertical transmission rate to saliva
FIR-S: Filial infective rate
RT-qPCR: Real-time RT-PCR
Pfu: Plaque-forming units
CPE: Cytopathic effect
2nd GC: Second gonotrophic cycle
VTR-H: Vertical transmission rate to head
FIR-H: Filial infection rate to head
LACV: La Crosse virus
TOT: Transovarial transmission
EVAg: European Virus Archive goes global
FFU: Focus-forming units
TCID_50_: Tissue Culture Infectious Dose_50_
DMEM: Dulbecco’s modification of Eagle’s medium
