# Supplementary material for: Vertically infected Aedes aegypti excrete infectious arboviruses in saliva
Source: BMC Biol. 2026 Feb 25;24:87. doi: 10.1186/s12915-026-02562-2 (PMC13040967; doi:10.1186/s12915-026-02562-2)
Supplement: Supplementary file 1 — Additional file 1: Table 1. Features of Zika, chikungunya, and dengue virus type 1 viral strains. [file 12915_2026_2562_MOESM1_ESM.docx]

Additional file 1: Table 1. Features of Zika, chikungunya, and dengue virus type 1 viral strains used in experimental blood meals provided to *Aedes aegypti* for vertical transmission assays

| **Viral strain**  (dose provided) | **Genotype** | **Year of first isolation/Place/host** | **Viral stock passage history** | **Cell type used for virus passage** | **GenBank accession number** |
| --- | --- | --- | --- | --- | --- |
| **ZIKV**  (10^7^ TCID50/mL) | African genotype | 1984/Senegal/*Aedes taylori* | P5 | Vero cells (ATCC, ref. CCL- 81) | KU955592 (complete genome) |
| **CHIKV**  (10^7^ TCID50/mL) | Asian genotype | 2014/Guadeloupe/human | P2 | Vero cells (ATCC, ref. CCL- 81) | OR488122 (complete genome) |
| **DENV-1**  (10^7^ FFU/mL) | genotype V | 2013/Guadeloupe/human | P2 | C6/36  ( ATCC, ref. CRL-1660) | OR486055 (complete genome) |

TCID50: median tissue culture infectious dose. FFU: focus-forming units.
