# Supplementary material for: Vertically infected Aedes aegypti excrete infectious arboviruses in saliva
Source: BMC Biol. 2026 Feb 25;24:87. doi: 10.1186/s12915-026-02562-2 (PMC13040967; doi:10.1186/s12915-026-02562-2)
Supplement: Supplementary file 2 — Additional file 2: Table 2. Nucleotide DENV-1 fragments obtained from vertically infected Aedes aegypti. [file 12915_2026_2562_MOESM2_ESM.docx]

Additional file 2: Table 2. Nucleotide DENV-1 fragments obtained from heads of vertically infected *Aedes aegypti* females

|  | **Nucleotide sequences** |
| --- | --- |
| **Head daughter 228’’R** | Fragment 1 (248 bp, *c* gen)  CGGAAGCTTGCTTAACGTAGTTCTAACAGTTTTTTATTAGAGAGCAG  ATCTCTGATGAACAACCAACGGAAAAAGACGGGTCGACCGTCTTTC  AATATGCTGAAACGCGCGAGAAACCGCGTGTCAACTGGTTCACAGT  TGGCGAAGAGATTCTCAAAAGGATTGCTTTCAGGCCAAGGACCCAT  GAAATTGGTGATGGCTTTCATAGCATTTCTAAGATTTCTAGCCATAC  CCCCAACAGCAGGAAT  Fragment 2 (187 bp, *e* gen)  TTGACTGCTGGTGCAATGCCACAGACACATGGGTAACCTATGGGAC  GTGTTCTCAAACCGGCGAACACCGACGAGAGAAACGTTCCGTGGCA  CTGGCCCCACACGTGGGACTTGGTCTAGAAACAAGAACCGAAACAT  GGATGTCCTCTGAAGGCGCTTGGAAACAAATACAAAGAGTGGAAAC  TTGGGCTTTGAGACACCCAGGATTCACGGTGATAGCCTTGTTTTTAG  CAC  Fragment 3 (205 bp, *e* gen)  GGGTAATGGCTGCGGACTATTCGGAAAAGGAAGTCTATTGACGTGT  GCCAAGTTCAAGTGTGTGACAAAACTAGAAGGAAAGATAGTTCAAT  ATGAAAACCTAAAATATTCAGTGATAGTCACTGTCCACACTGGGGA  CCAGCACCAGGTGGGAAACGAGACCACAGAACATGGAATAATTGCA  ACCATAACACCTCAAGCTCCC |
| **Head daughter 258”K** | Fragment 1 (244 bp, *c* gen)  AGCTTGCTTAACGTAGTTCTAACAGTTTTTTATTAGAGAGCAGATCTC  TGATGAACAACCAACGGAAAAAGACGGGTCGACCGTCTTTCAATATG  CTGAAACGCGCGAGAAACCGCGTGTCAACTGGTTCACAGTTGGCGAAGAGATTCTCAAAAGGATTGCTTTCAGGCCAAGGACCCATGAAATTGGTGATGGCTTTCATAGCATTTCTAAGATTTCTAGCCATACCCCCAACAGCAGGAAT  Fragment 2 (233 bp, *e* gen)  GTCGCCGAACGTTTGTAGACAGAGGCTGGGGTAATGGCTGCGGACTATTCGGAAAAGGAAGTCTATTGACGTGTGCCAAGTTCAAGTGTGTGACAAAACTAGAAGGAAAGATAGTTCAATATGAAAACCTAAAATATTCAG  TGATAGTCACTGTCCACACTGGGGACCAGCACCAGGTGGGAAACGAGACCACAGAACATGGAATAATTGCAACCATAACACCTCAAGCTCCC |
