# Supplementary material for: Vertically infected Aedes aegypti excrete infectious arboviruses in saliva
Source: BMC Biol. 2026 Feb 25;24:87. doi: 10.1186/s12915-026-02562-2 (PMC13040967; doi:10.1186/s12915-026-02562-2)
Supplement: Supplementary file 3 — Additional file 3: Fig. S1. Standard curves for dengue virus type 1, chikungunya, and Zika viruses. [file 12915_2026_2562_MOESM3_ESM.docx]

Additional file 3: Fig. S1.


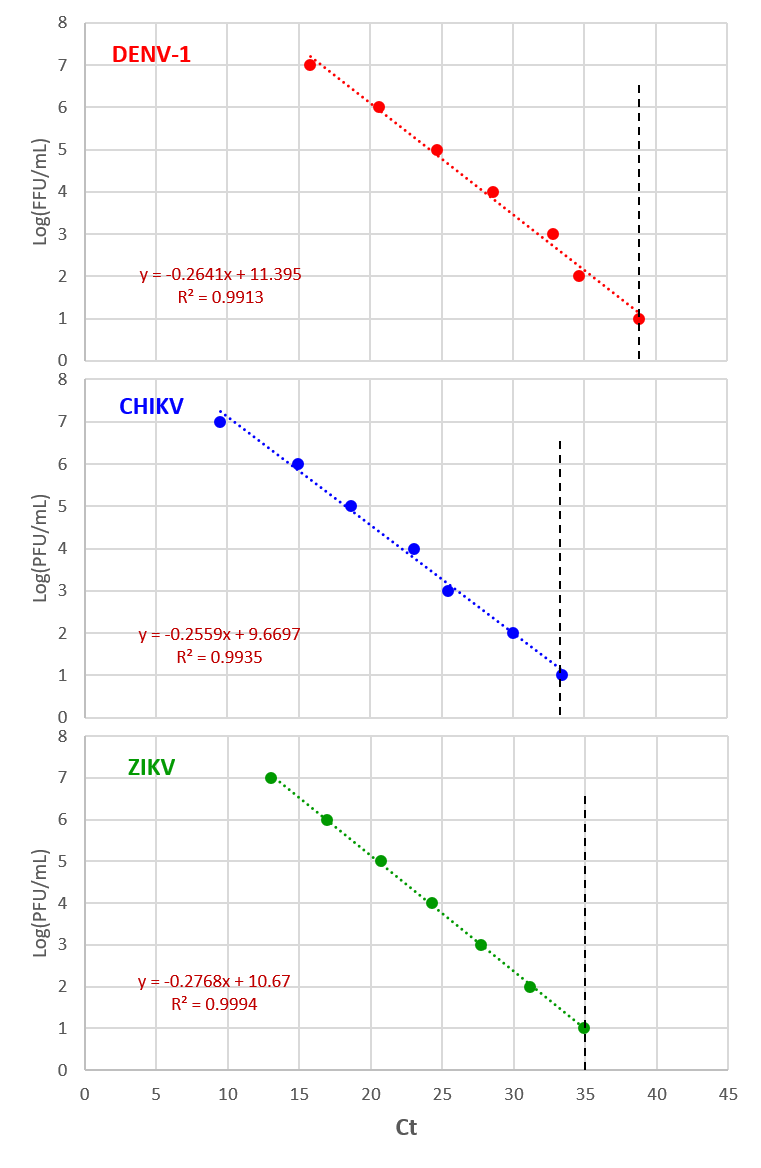


Additional file 3: Fig. S1. Standard curves for dengue virus type 1 (red line), chikungunya (blue line), and Zika viruses (green line) using the same viral strains employed in the vertical transmission assays. Black vertical line points out the Ct value selected for each virus.
