# Supplementary material for: Vertically infected Aedes aegypti excrete infectious arboviruses in saliva
Source: BMC Biol. 2026 Feb 25;24:87. doi: 10.1186/s12915-026-02562-2 (PMC13040967; doi:10.1186/s12915-026-02562-2)
Supplement: Supplementary file 4 — Additional file 4: Table 3. Sequences of primers used for specific amplification of CHIKV. [file 12915_2026_2562_MOESM4_ESM.docx]

**Additional file 4: Table 3. Sequences of primers used for specific amplification of CHIKV in *Aedes aegypti* samples from vertical transmission assays before sequencing**

| Primer name (primer numbering according to position of its 5’_ base on MW281311.1 sequence) | Sequence (5’-3’) | Amplification size (bp) |
| --- | --- | --- |
| Chik-1S | ATGGCTGCGTGAGACACAC | 1487 |
| Chik-1488R | TGTATGGGATCAGGTCGGTT |  |
| Chik-1332S | GCAGAAAACACACACGGTCT | 1811 |
| Chik-3143R | TGAGACCACTGCCTATCATTTA |  |
| Chik-3067S | GACCTTCGATACATTCCAAA | 1505 |
| Chik-4572R | CACGCGAACAATATCGCAGT |  |
| Chik-4546S | CAAGTAGAGCTGCTGGATGA | 1756 |
| Chik-6302R | CACGTTGAATACTGCTGAGT |  |
| Chik-6224S | ACACACTACAGAATGTACTGGCA | 1487 |
| Chik-7711R | TGGCTTCTGTTGGGGTACCG |  |
| Chik-7673S | CCAGCTGATCTCAGCAGTTA | 1204 |
| Chik-8877R | CGTACATGAGTGACTAATCTTCCT |  |
| Chik-8797S | CAGCACCGTGTACGATTACTGG | 1680 |
| Chik-10477R | TGTCGAAAGGTGTCCAGGCTG |  |
| Chik-10390S | GAAATAACATCACTGTAACTGCC | 880 |
| Chik-11270R | GCCTGCTGAACGACACGCAT |  |
