# Supplementary material for: Vertically infected Aedes aegypti excrete infectious arboviruses in saliva
Source: BMC Biol. 2026 Feb 25;24:87. doi: 10.1186/s12915-026-02562-2 (PMC13040967; doi:10.1186/s12915-026-02562-2)
Supplement: Supplementary file 5 — Additional file 5: Table 4. Sequences of primers used for specific amplification of ZIKV. [file 12915_2026_2562_MOESM5_ESM.docx]

**Additional file 5: Table 4. Sequences of primers used for specific amplification of ZIKV in *Aedes aegypti* samples from vertical transmission assays before sequencing**

| Primer name (primer numbering according to position of its 5’_ base on KJ776791.2 sequence) | Sequence (5’-3’) | Amplification size (bp) |
| --- | --- | --- |
| ZIKV- 1S | AGTTGTTGATCTGTGTGAGTCAG | 939 |
| ZIKV-940R | AATCAGCAGTATCATGACCAAGT |  |
| ZIKV-882S | TTAGCAGCAGCTGCCATCGC | 1001 |
| ZIKV-1883R | GGTACACARGGAGTATGACACG |  |
| ZIKV-1785S | GCTGGAGCTCTGGAGGCTG | 958 |
| ZIKV-2743R | ATCCCACAACGACCGTCAGTT |  |
| ZIKV-2708S | GGAGCTCAACGCAATCCTGGA | 1116 |
| ZIKV- 3824R | TGTCCAATTAGCTCTGAAGATG |  |
| ZIKV-3581S | AGTGCTTGTGATTCTGCTCATGGT | 990 |
| ZIKV-4571R | GTACCACGCTCCAGCTGCA |  |
| ZIKV-4525S | TGGTCCTGATGACCATCTGTG | 1075 |
| ZIKV-5600R | GGTGTCCATAATTGGTGAGTTG |  |
| ZIKV-5422S | TACTACAGCCAATYAGAGTCC | 1107 |
| ZIKV-6529R | CGAGGTTGTCAATGGCTTCCT |  |
| ZIKV-6388S | CGAGGTGGATGGAYGCCAGAG | 1124 |
| ZIKV-7512R | CACAAAGTGGAAGTTGCSGCTGT |  |
| ZIKV-7329S | ACGGCAGCTGGCATCATGAAG | 886 |
| ZIKV-8215R | TGCTGGTGTATGGGCACAACA |  |
| ZIKV-8138S | AGAAGCACGGACGCTCAGAG | 1005 |
| ZIKV-9143R | CATCCAGTGATCCTCGTTCAAG |  |
| ZIKV-8935S | CAGTGGAAGCTGTGAACGATC | 1375 |
| ZIKV-10310R | GTGGATAGGTARTCCATGTAC |  |
| ZIKV-10220S | TCTCATAGGGCACAGACCGC | 422 |
| ZIKV-10642R | TCCCTCTTCTGGAGATCCAC |  |
